# Nationwide Temporal and Geographical Distribution of Tick Populations and Phylogenetic Analysis of Severe Fever with Thrombocytopenia Syndrome Virus in Ticks in Korea, 2020

**DOI:** 10.3390/microorganisms9081630

**Published:** 2021-07-30

**Authors:** Min-Goo Seo, Byung-Eon Noh, Hak Seon Lee, Tae-Kyu Kim, Bong-Goo Song, Hee Il Lee

**Affiliations:** Division of Vectors and Parasitic Diseases, Korea Disease Control and Prevention Agency, 187 Osongsaenmyeong2-ro, Heungdeok-gu, Cheongju, Chungbuk 28159, Korea; koreasmg@korea.kr (M.-G.S.); nbudia@korea.kr (B.-E.N.); hslee8510@korea.kr (H.S.L.); tkkim80@korea.kr (T.-K.K.); gign1204@korea.kr (B.-G.S.)

**Keywords:** tick, SFTS, temporal, geographical, phylogenetic analysis, zoonosis, vector-borne

## Abstract

Since 2010, the Korea Disease Control and Prevention Agency has established centers at 16 locations to monitor disease vectors and pathogens. Here, we examined tick populations to understand the geographical and temporal distribution of severe fever with thrombocytopenia syndrome virus (SFTSV) vectors in 2020. From April to November, 63,376 ticks were collected from traps to monitor tick populations, with a trap index of 41.3. Tick incidence varied from April to October, with population peaks observed for nymphs in May, adults in July, and larvae in September. The predominant tick species were *Haemaphysalis longicornis*, *Haemaphysalis* spp., *H. flava, Ixodes* spp., *Amblyomma testudinarium,* and *Ixodes nipponensis*. Approximately 50% of the collected ticks were pooled into 2973 groups to detect the rate of SFTSV infection in ticks. The minimum infection rate (MIR) of SFTSV was 0.2%, and Andong had the highest MIR for SFTSV (4.0%). The B3 genotype was the most prevalent (52.2%) followed by B2 (28.6%), B5 (15.9%), B4 (1.6%), and B6 (1.6%). We identified widely distributed tick species and a high degree of diversity in SFTSV strains in ticks from different geographical regions. The results may provide a basis for future epidemiological studies and risk assessments for tick-borne diseases.

## 1. Introduction

Severe fever with thrombocytopenia syndrome (SFTS) is an emerging zoonotic tick-borne infectious disease; the SFTS virus (SFTSV), recently renamed *Huaiyangshan banyangvirus*, belongs to the genus *Banyangvirus* in the family *Phenuiviridae* [1]. The SFTSV genome contains negative-stranded RNA segments, including the large (L) segment, which encodes the RNA-dependent RNA polymerase; the medium (M) segment, which encodes the envelope glycoproteins Gc and Gn; the small (S) segment, which harbors a nucleoprotein; and a nonstructural S segment protein, which is encoded by an ambisense approach [2]. SFTSVs can be crudely classified into six genotypes (A–F) based on the classification method that was previously used in China [3]. In a previous study, the majority of Korean SFTSV isolates were classified as genotype B, which was divided into at least three different sub-genotypes (B1, B2, and B3) [4].

SFTS is known to be transmitted by several ticks [5,6], and ticks of the family Ixodidae are regarded as vectors of SFTSV. The role of ticks as vectors of SFTS and the proportion of infected individuals are crucial tools for appropriate tick-borne disease control programs. The prevalence of SFTS in ticks is an important tool for studying the epidemiology of tick-borne diseases. This virus has also been detected in reptiles, small mammals such as shrews and rodents, and domestic animals such as dogs, cats, cattle, and sheep [2]. The prevalence of SFTS patients in Korea has been recorded as 165 in 2016, 272 in 2017, 259 in 2018, 223 in 2019, and 243 in 2020 [7]. In 2020, the regional SFTS cases per 100,000 people were 20 in Hwacheon, 11 in Gapyeong, 9 in Inje, and 5 in Pocheon in the northern area; 7 in Gongju, 6 in Goryeong, and 6 in Yeongyang in the central area; and 13 in Hapcheon, 11 in Imsil, 9 in Jangsu, 9 in Hadong, and 8 in Muju in the southern area. In Korea, annual SFTS cases generally rise in April and decline in November. A previous study reported October as having the highest number of cases followed by August [7].

To date, the climate of Korea has steadily changed from a temperate to a subtropical area; this environmental change might make the Korean Peninsula a prime habitat for explosions in tick populations in the near future. Ticks depend on environmental conditions for their survival and benefit from climate change [8]. Since 2010, the Korea Disease Control and Prevention Agency has established regional centers to monitor the seasonal prevalence of disease vectors and their associated pathogens related to climate change [9]. Due to the increasing number of SFTS infections in humans, understanding the population dynamics of ticks is becoming increasingly important to public health. Therefore, we studied the temporal and geographical distribution of tick populations and the epidemiology of SFTSV in ticks in Korea. These results could be used to estimate the source of infections in future epidemiological research and could provide risk assessments for vector-borne pathogens based on the effects of global warming.

## 2. Materials and Methods

### 2.1. Ethical Approval

The animal protocol used in this study was reviewed and approved according to the guidelines for ethical procedures and scientific care by the Institutional Animal Care and Use Committee of the Korea Centers for Disease Control and Prevention (KCDC-093-18). Specific permission for each collection site was not required because the sites were not located within national parks or protected areas. The collected ticks did not include endangered species.

### 2.2. Tick Collection and Species Identification

**Figure 1:**
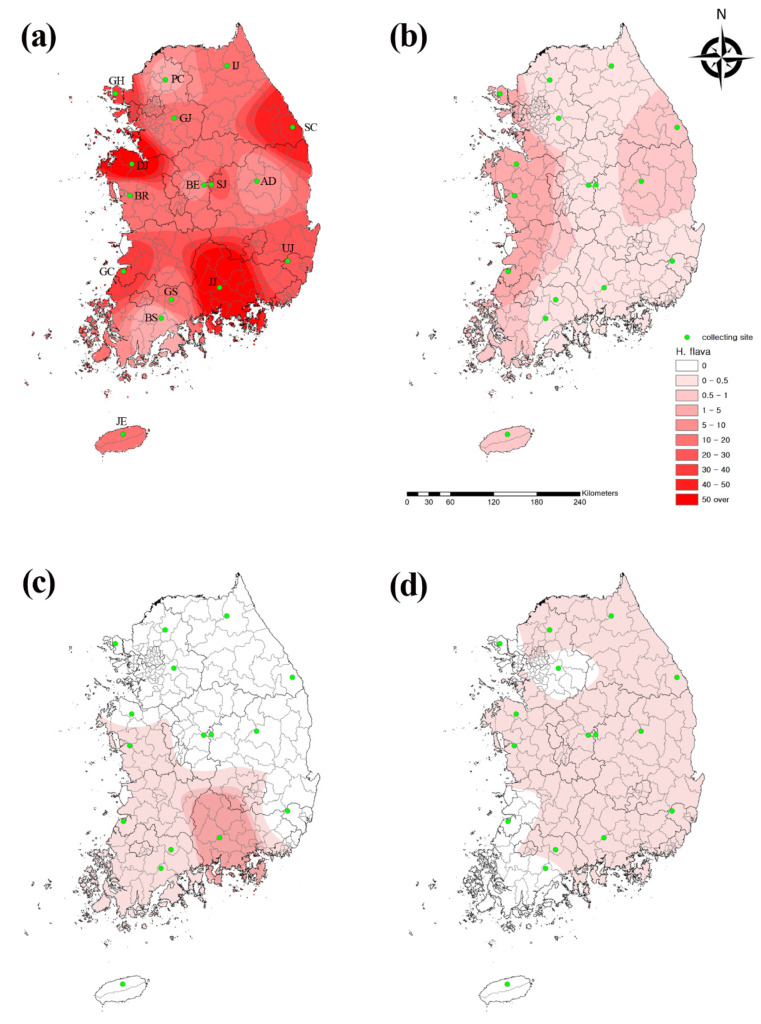
Geographical distribution of four tick species. (**a**) *Haemaphysalis* *longicornis,* (**b**) *H.* *flava,* (**c**) *Amblyomma testudinarium,* and (**d**) *Ixodes nipponesis* collected from traps at 16 collection sites nationwide in Korea in 2020. The map color indicates the trap index (TI; 0 to >50). Collection traps are denoted by green dots. The TI shows the number of ticks per trap. PC, Pocheon; IJ, Inje; GH, Ganghwa; SC, Samcheok; GJ, Gwangju; DJ, Dangjin; BR, Boryeong; BE, Boeun; AD, Andong; SJ, Sangju; GC, Gochang; UJ, Ulju; GS, Gokseong; BS, Boseong; JJ, Jinju, JE, Jeju.

Ticks were captured and collected from traps at 16 collection sites nationwide, which are located in the northern (Pocheon, Inje, Ganghwa, Samcheok, and Gwangju), central (Dangjin, Boryeong, Boeun, Andong, and Sangju), and southern (Gochang, Ulju, Gokseong, Boseong, and Jinju) areas as well as on the southernmost Island (Jeju) (Figure 1), in 2020. The collections were performed at each location once a month, in the period from April to November. Each collection site consisted of four different environments and contained three traps per environmental point. These environments are all very common in rural areas of Korea and consist of grassland, grave, grass thicket, and mountain roads.

**Figure 2:**
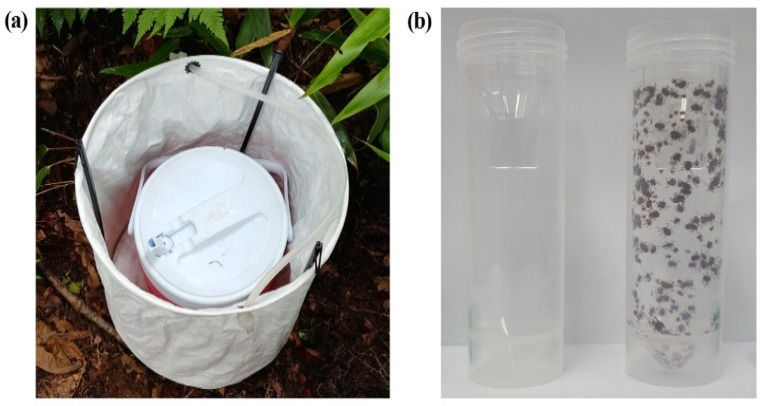
Dry ice bait collecting trap (**a**), which consists of a tarpaulin cylindrical trap and a cylindrical ice chest, installed at each environmental point and (**b**) a tick collection tube that prevents the escape of ticks.

In total, 12 dry ice bait-collecting traps (Shin-Young Commerce System, Gyeonggi, Korea) were set up at each point at the collection sites at 10 m intervals (three traps per environmental point). Inside the tarpaulin cylindrical trap (35 cm diameter × 40 cm height), a cylindrical ice chest (10 cm diameter × 30 cm height) containing four pieces of dry ice (approximately 2.5 kg) was placed with its flip-top spout open to lure ticks overnight (Figure 2a). Ticks that were either inside or on the surface of the traps were collected into the tick collection tube (Patent no. 10-0925882) (Figure 2b) the following day using either fine forceps or an aspirator (Bioquip, Rancho Dominguez, CA, USA). Ticks were identified both taxonomically and by life stage via optical microscopic examination using morphological keys [10]. Adults and nymphs were identified to the species level, while larvae were identified to the genus level due to morphological similarities.

### 2.3. Molecular Detection of SFTSV from Ticks

Approximately half of the collected ticks were used to detect SFTSV infection, and the remainder were stored in 70% ethanol for long-term use as a biological resource. The tick pools (1–5 for adults, 1–30 for nymphs, and 1–50 for larvae) were homogenized by species, survey period, and collection site using a Precellys^®^ CK28-R Lysing kit (bead tube for hard tissue homogenization; Bertin Technologies, Bretonneus, France) and Precellys^®^ evolution (homogenizer; Bertin Technologies, Montigny-le-Bretonneux, France) followed by total RNA extraction from the pools using a commercial Direct Zol^TM^ RNA extraction kit (Zym Research, Irvine, CA, USA) with TRIzol reagent (Invitrogen, Carlsbad, CA, USA) according to the manufacturers’ instructions.

To detect the partial SFTSV M-segmented gene, a one-step reverse transcription PCR (RT-PCR) was performed using a Diastar^TM^ 2× OneStep RT-PCR premix (SolGent Co., Daejeon, Korea) with SFTSV-specific MF3 (5’-GATGAGATGGTCCATGCTGATTCT-3’)/MR2 (5’-CTCATGGGGTGGAATGTCCTCAC-3’) primers [3]. Afterwards, nested PCR was performed using an AccuPower HotStart PCR Premix Kit (Bioneer, Daejeon, Korea) with SFTSV-specific MMF3 (5’-TAAACTTGTGTCGTGCAGGC-3’)/MMF2 (5’-CCCAGCGACATCTCCTTACA-3’) primers [3]. The minimum infection rates (MIRs, number of positive pool of mites/total number of ticks tested × 100) were then calculated.

### 2.4. DNA Sequencing and Phylogenetic Analysis

Purified PCR products were obtained using forward and reverse PCR amplification primers and were then sequenced by Macrogen (Seoul, Korea). Each raw chromatogram of both the forward and reverse sequences were visually inspected to detect double peaks and were combined into a final whole sequence using the CLC Main Workbench 6.9 (CLC Bio, Qiagen, Aarhus, Denmark). Sequences were analyzed using the multiple sequence alignment program CLUSTAL Omega (version 1.2.1, http://www.clustal.org/omega/, accessed on 1 April 2021). The sequence alignment results were modified using BioEdit (version 7.2.5, http://www.mbio.ncsu.edu/BioEdit/bioedit.html, accessed on 1 April 2021) and were analyzed using a similarity matrix. Phylogenetic analysis was performed with MEGA (version 6.0, https://www.megasoftware.net/, accessed on 1 April 2021) using the maximum likelihood method, and tree stability was assessed using bootstrap analysis with 1000 replicates.

### 2.5. Geographical Analysis

Distribution maps were drawn by interpolation using the inverse distance weighted (IDW) technique among spatial analyst tools in ArcGIS 9.0 (2004, Environmental Research Systems Institute, Redlands, CA, USA) to compare the geographical tick distribution.

## 3. Results

### 3.1. Prevalence of Tick Populations

In total, 1536 traps were installed and 63,376 ticks (41.3 Trap index (TI), number of collected ticks/number of installed traps) representing three genera and five species were collected, including *H. longicornis, H. flava, H. japonica*, *Haemaphysalis* spp., *I. nipponensis*, *Ixodes* spp., and *A. testudinarium*. Among the collection sites, Samcheok (18.7%, 11,861/63,376; TI, 123.6) and Pocheon (0.8%, 534/63,376; TI, 5.6) had the highest and lowest numbers of collected ticks, respectively (Table 1). Samcheok (18.7%), Dangjin (14.3%), Jinju (12.8%), and Jeju (10.3%) accounted for more than half of the ticks collected. Among the tick species, *H. longicornis, H. flava,* *I. nipponensis,* and *Haemaphysalis* spp. were evenly distributed throughout the country, whereas *A. testudinarium* and *Ixodes* spp. were mostly distributed in the Jinju (71%) and Dangjin (99.3%) regions, respectively, and *H. japonica* was only found in the Inje region (n = 2 ticks).

Among the adult and nymph ticks, *H. longicornis* (96.5%, 35,943/37,240; TI, 23.4) was the most prevalent tick species followed by *H. flava* (2.8%, 1049/37,240; TI, 0.7), *I. nipponensis* (1.7%, 631/37,240; TI, 0.04), *A. testudinarium* (0.5%, 183/37,240; TI, 0.1), and *H. japonica* (0.005%, 2/37,240; TI, 0.001). All of the collected larval ticks were identified as either *Haemaphysalis* spp. (98.3%, 25,688/26,136; TI, 16.7) or *Ixodes* spp. (1.7%, 448/26,136; TI, 0.3) (Table 2) due to morphological similarities at the species level. No *Amblyomma* larvae were collected. The numbers of *H. longicornis*, *Haemaphysalis* spp., *H. flava, Ixodes* spp., *I. nipponensis*, and *A. testudinarium* were the highest in May (38.7%, 13,925/35,943), September (48.7%, 12,517/25,688), September (21.3%, 223/1049), July (96.7%, 433/448), July (28.6%, 18/63), and May (48.6%, 89/183), respectively. However, the population density of *H. japonica* was too low to assess temporally.

**Figure 3:**
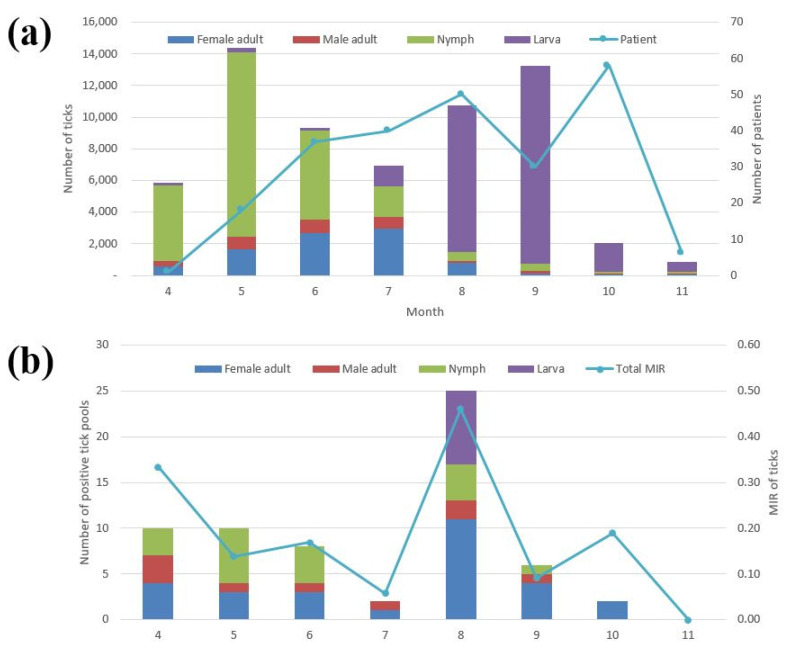
Temporal distribution in the populations of total ticks and SFTS patients (**a**) and the number of positive tick pools and MIR of ticks (**b**) collected from traps in Korea from April to November in 2020. MIR, minimum infection rate (number of positive pools of ticks/total number of ticks tested × 100).

Among the tick stages, larvae (41.2%, 26,136/63,376; TI, 17) had the highest incidence followed by nymphs (39.8%, 25,226/63,376; TI, 16.4), female adults (14.1%, 8915/63,376; TI, 5.8), and male adults (4.9%, 3099/63,376; TI, 2) (Table 2). The numbers of female adults, male adults, nymphs, and larvae were the highest in July (33.2%, 2959/8915), May (27.4%, 849/3099), May (46.2%, 11,643/25,226), and September (47.9%, 12,518/26,136), respectively (Figure 3a). Temporally, the highest peak in tick incidence was observed in May (22.7%, 14,371/63,376; TI, 74.8), when nymphs were highly prevalent; a second peak was observed in September (20.9%, 13,265/63,376; TI, 69.1), when larvae were highly prevalent. In addition, SFTS cases began to appear in April and ended in November. The highest number of cases were reported in October followed by August, July, June, September, May, November, and April. (Figure 3a). Among the different environments, grassland (35.8%, 22,672/63,376) had the highest incidence of ticks among all of the trapping points followed by mountain roads (24.2%, 15,367/63,376), graves (22.1%, 14,012/63,376), and grass thicket (17.9%, 11,325/63,376).

We analyzed the collected data using the IDW interpolation method in ArcGIS to assess the geographical distribution of chigger mites in Korea (Figure 1). We found that *H. longicornis*, *H. flava*, and *I. nipponesis* were mostly distributed nationwide, whereas *A. testudinarium* was found only in the southern and western areas of the country.

### 3.2. Prevalence of SFTSV in Ticks

Among the 63,376 ticks that were sampled, 32,052 ticks (~50%) were pooled into 2973 pools for SFTSV infection monitoring. The MIR of SFTSV was 0.2% (63 pools/32,052 ticks; Table 3). The ticks collected from the Andong (4.0%, 16/399) region had the highest MIR of SFTSV followed by Gwangju (1.9%, 18/961), Gokseong (1.6%, 18/1104), Gochang (0.5%, 10/1825), and Samcheok (0.02%, 1/5944) (Table 3).

Among the tick species, *I. nipponensis* (3.9%, 2/51) had the highest MIR of SFTSV followed by *H. flava* (2.6%, 16/618), *A. testudinarium* (1.9%, 2/106), *H. longicornis* (0.2%, 35/18,193), and *Haemaphysalis* spp. (0.1%, 8/12,856). Among the tick stages, female adults (0.61%, 28/4558) had the highest MIR of SFTSV followed by adult males (0.55%, 9/1628), nymphs (0.14%, 18/12,784), and larvae (0.06%, 8/13,082). Among the different environments, the graves (0.3%, 22/7097) had the highest MIR of SFTSV followed by grassland (0.2%, 22/11,427), mountain roads (0.1%, 11/7766), and grass thicket (0.1%, 8/5762).

The highest number of positive tick pools were observed in August (39.7%, 25/63; MIR, 0.5%) followed by April (15.9%, 10/63; MIR, 0.3%), May (10/63; MIR, 0.14%), June (12.7%, 8/63; MIR, 0.17%), September (9.5%, 6/63; MIR, 0.09%), July (3.2%, 2/63; MIR, 0.06%), and October (3.2%, 2/63; MIR, 0.19%); no SFTSV-positive ticks were observed in November (Figure 3b).

### 3.3. Molecular and Phylogenetic Analyses

**Figure 4:**
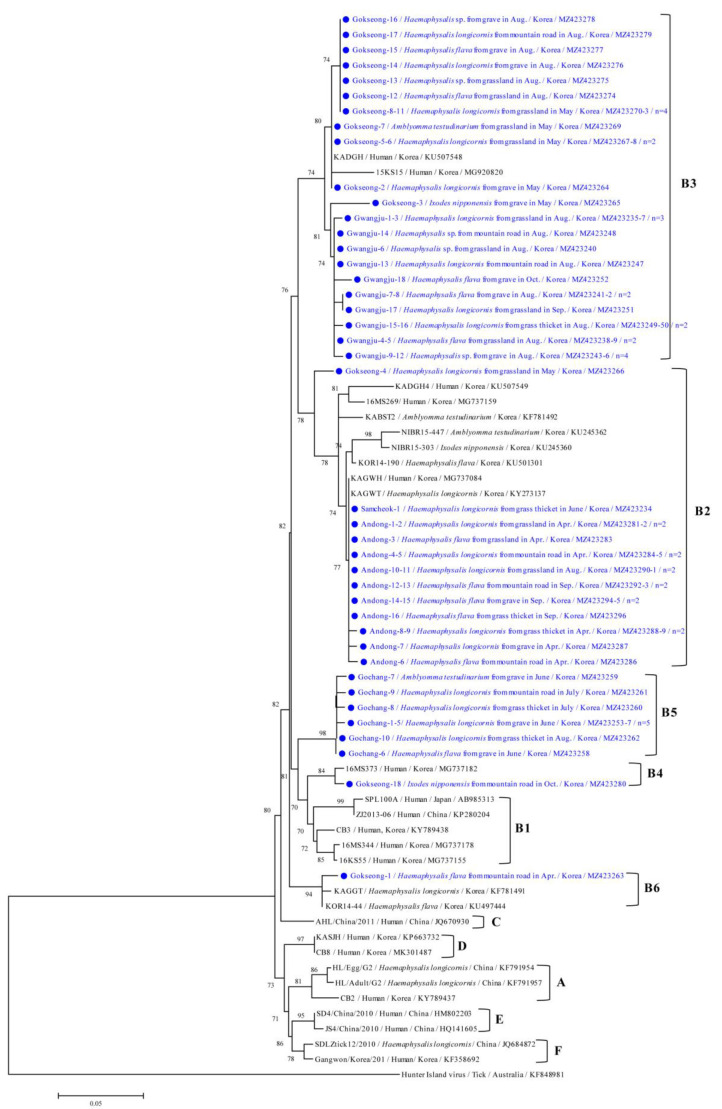
Phylogenetic tree of SFTSV based on M segment gene sequences. The maximum likelihood method was used to create the tree; closed circles and blue letters indicate the sequences detected in this study. The GenBank accession numbers are shown, and the SFTSV genotypic groups are indicated by the letters to the right of the parentheses. Branch numbers indicate bootstrap support levels (1000 replicates), and the scale bar displays the substitution numbers for each nucleotide.

Phylogenetic analysis showed that the SFTSV M segment (Figure 4) was clustered with previously documented sequences and was divided into five groups of the B genotype. The B3 genotype (52.4%, 33/63) was the most common followed by B2 (28.6%, 18/63), B5 (15.9%, 10/63), B4 (1.6%, 1/63), and B6 (1.6%, 1/63).

The 33 B3 strains, 18 B2 strains, and 10 B5 strains found in this study shared 97.5%–100%, 96.6%–100%, and 99.0%–100% identity, respectively. Each sequence shared 95.1%–100%, 94.7%–100%, and 96.1%–100% identity, respectively, with previously reported SFTSV strain isolates from GenBank. The representative sequences reported in the present study have been submitted to GenBank under the following accession numbers: MZ423234-MZ423296 (SFTSV M segment).

## 4. Discussion

Traditional surveillance methods for ticks, including the flagging method, in which the vegetation is swept with a flannel cloth, are primarily used to harvest questing ticks [11,12]. However, the flagging method is susceptible to sampling mistakes stemming from vegetation types, the experience of the sampler, and the spatial distribution of ticks [11]. In a previous study, to evaluate the effects of habitat on surveillance method effectiveness, four methods (dry ice, CO_2_ flagging, CO_2_ dragging, and dragging) were compared in four habitat types [13]. In the habitat comparison, the dry ice trap collection method was the most effective in upland deciduous and coniferous habitats. Since dry ice-baited traps were the most reliable across habitat types, this technique is preferred when sampling areas that may undergo changes in vegetation, such as those subject to periodic prescribed fires, or when comparing habitat types. In most cases, the use of dry ice traps is less time-consuming at each location, which can reduce the amount of time that samplers are exposed to potentially infected ticks. In addition, the traps are relatively easy to maintain because they are stationary, which makes them undeterred by vegetation density and type, and they do not require expertise [13]. Therefore, we used dry ice bait-collecting traps, which may be less error-prone than flagging. To monitor the temporal and geographical distribution of tick populations, we collected unfed ticks from traps in rural environments that are relevant to human activities, as such activities are associated with an increased incidence of SFTS. Per our collection trap results, grassland is a more favorable tick habitat than grass thicket. The prevalence of tick species in the grassland areas found in our study may be associated with the high incidence of SFTSV infection in the inhabitants of rural areas in Korea because most agricultural activities occur in agricultural fields and the nearby grasslands [11].

Questing ticks from our collection traps were analyzed in monthly surveys from April to November 2020. We identified the geographical and temporal distribution of tick species and found that in 2020, the majority of ticks were collected in the Samcheok (18.7%) and Dangjin (14.3%) regions. The Samcheok (19.1%) and Dangjin (18.9%) regions also showed a high incidence of ticks in 2019 [14]. Thus, we conclude that these regions have a higher geographical distribution of tick populations than other regions. Geographical differences in tick distribution may be affected by environmental and ecological factors. The mechanisms underlying the increase in SFTS prevalence remain uncertain, but the spread of emerging zoonotic viruses are normally attributed to mechanisms such as increased contact between human and wildlife populations and the geographical spread of arthropod vectors or their vertebrate hosts outside of endemic areas [15]. Tick incidence differed by both geographical area and collection period, with the most prevalent being the Jinju region in April (30.3%), the Dangjin region in July (36.3%), the Samcheok region in August (32.2%), the Ulju region in October (46.3%), and the Jeju region in November (61%).

In Korea, peaks in adult, nymph, and larval tick incidence are normally observed from June to August, May to June, and August to September, respectively [8,11]. Adult ticks lay eggs in the summer, after which the larvae molt into the nymph stage in the autumn. Therefore, the larvae population density increased from August to September. Ticks are mainly in the nymph stage over the winter [16], resulting in increased nymph population during the spring season. In this study, the primary tick incidence varied from April to October, with the highest peaks in the population observed in nymphs in May, adults in July, and larvae in September 2020. 

We compared the temporal distribution between the tick population and the incidence of SFTS in patients. Nymph populations began to increase from April to May, and this corresponds to the slightly delayed increase in the number of patients with SFTS from May to June [7]. In addition, the high incidence of SFTS observed from July to August is generally due to the increase in the adult tick population that peaks from June to July. Due to the sudden decrease in adult tick populations in August, the number of patients with SFTS is low in September. Furthermore, because of the increase in larval tick populations from August to September, the highest incidence of SFTS is observed in October. This phenomenon may be related with the Korean Thanksgiving holiday that occurs during this period (i.e., the beginning of October in 2020). During this holiday, people visit the graves of their ancestors to trim vegetation, clean the nearby area, and offer drinks and food to their ancestors. Therefore, we propose that humans are exposed to ticks in the course of weeding graves during the Korean Thanksgiving period [17]. Finally, the sudden decrease in the total tick population in October corresponds to the reported decrease in the incidence of SFTS patients in November. This result indicates that temporal high densities of each tick stage might affect the incidence rate of SFTS in each season. This study assessed the correlation between the appearance of ticks and the incidence of patients, analyzed at one-month intervals from the first tick collection date to outbreaks SFTS in patients. We expected that the geographical regions where SFTS is highly endemic would show higher tick populations; however, we were unable to determine any correlation between the relative rates of SFTS and TI. For example, the Pocheon region, which had the highest number of reported cases in 2020 (n = 7), had the lowest TI (5.6) compared to the highest TI (123.6) in the Samcheok region, where only two cases have been reported. Furthermore, we compared the geographical distribution of SFTSV infection in ticks and the incidence of SFTS in patients but were unable to find a relationship. Although ticks that harbor SFTSV are known to be distributed throughout the country, this fact does not relate to geographical patient incidence [17]. For example, the Pocheon and Jinju (7 and 7 patients, respectively) regions showed no SFTS incidence. An exception was the Andong and Gwangju regions, which had no reported SFTS cases while also having the highest MIR (4.0% and 1.9%, respectively). The differences in the occurrence of SFTS cases, tick populations, and SFTSV infection in ticks may explain why humans are incidental tick hosts. Large human populations and their companion animals are exposed to all tick life stages and their related zoonotic pathogens when conducting outdoor activities in Korea, including: (1) recreational hiking in forested mountains and other leisure activities in areas with grasses and other vegetation; (2) military training activities; (3) outdoor construction; (4) tending to graveyards during the Korean Thanksgiving period, (5) forestry activities, and (6) cultivation [18,19,20]. Thus, further studies are needed to analyze the infection rate of SFTSV in ticks, the population densities of ticks, and the SFTS cases in patients. Continuous monitoring is needed to assess the patterns of tick and patient infections.

Furthermore, we performed molecular detection and a phylogenetic analysis of SFTSV in ticks. No SFTSV positivity in ticks was observed in November. Generally, SFTSV positivity is higher in adult ticks than in nymphs and larvae, except in May and June. In addition, infected adult ticks were detected during all periods, implying that adult ticks carrying SFTSV are more prevalent than nymphs and larvae because they have a higher chance of meeting a SFTV-infected host. Temporally, infected larval ticks were only detected in August, while the number of positive tick pools in adults was the highest. The number of positive tick pools in nymphs was the highest in May. In October, the MIR of SFTSV in ticks was relatively high, whereas the total number of positive tick pools was relatively low (only two adult ticks); however, the incidence of SFTS was the highest. This phenomenon may be explained by the high risk of SFTS infection in October caused by a smaller tick population with a higher infection rate. Thus, people should exercise increased caution when conducting outdoor activities during this period.

Transstadial and transovarial transmission are potential modes for the maintenance of SFTSV, as all stages of unfed ticks were found to be positive for SFTS [19]. In this study, *H. longicornis* (35 pools; 19 female adults, 7 male adults, and 9 nymphs) was the most prevalent tick species found to be harboring SFTSV followed by *H. flava* (16 pools; 7 female adults, 2 male adults, and 7 nymphs), *Haemaphysalis* spp. (8 pools; 8 larvae), *I. nipponensis* (2 pools; 2 female adults), and *A. testudinarium* (2 pools; 2 nymphs). In other studies in Korea, SFTSV was nationally detected in *H. longicornis* (0.46% MIR) from March to October between 2011 and 2012 [21]; *H. longicornis* (0.1% MIR) and *H. flava* (1.4%, MIR) in 2013 [12]; *H. longicornis* (3.8% MIR) from January to February in 2019 [16]; *H. longicornis* (5.7% MIR), *A. testudinarium* (23.5%, MIR), and *I. nipponensis* (13.3%, MIR) from humans from May to October in 2013 [5]; and *H. longicornis* (4.8%), *A. testudinarium* (20%), and *H. flava* (1.2%) from July to November in 2015 in national parks [6]. As the detection of SFTSV in a tick species does not prove the capacity of the tick species to act as a competent vector, additional research is needed to establish whether any of these other species are SFTS vectors. Furthermore, the infection and transmission capacity of the SFTSV is determined by the ability of the tick species to sufficiently amplify and spread the virus to humans and animals [15].

Korean SFTSVs in patients can be roughly classified into six genotypes (A–F) based on the classification method formerly used in China [3]. Inconsistent phylogenetic clustering segments showed that at least six different genotypes with nine different reassortments were co-circulating in Korea. This suggests that SFTSV strains undergo evolution in nature through re-assortment, resulting in the formation of new genotypes. In Korea, the majority of Korean SFTSV isolates were classified as genotype B (69.2%), which was divided into at least three different sub-genotypes (B1, B2, and B3); the most prevalent genotype was B2 (36.1%) followed by B3 (21.1%), and B1 (12%) [4]. In addition, the A (7.5%), F (6.8%), and D (3.8%) genotypes [4] and a minor portion of the C and E genotypes [22] were detected in patients in Korea. The most prevalent genotype in Japan was B2, and the total genotype distribution was similar to that seen in Korea, although genotypes A and F were not reported there. The SFTSV genotypes of China varied with 14 reported genotypes, the most prevalent being genotype F. In this study, the M gene showed five groups of B genotype variations in SFTSV strains from different regions. In Korea, various genotypes have been detected in humans, whereas in this study, we only detected the B genotype in ticks. This may have occurred due to a primer design that is too short (245 bp) to detect various genotypes; thus, we might have incidentally only detected the B genotype in ticks. Further studies are needed to design longer and more suitable primers that will allow the detection of other SFTSVs genotypes in ticks. We confirmed that the B3 genotype was the most common in ticks in Korea in 2020, and it was detected in the Gwangju and Gokseong regions. The B2 genotype was detected in the Gokseong, Samcheok, and Andong regions. The B1 sub-genotype was not detected in this study. In a previous study, the isolates that were used were human samples were divided into three sub-genotypes of the B genotype [4]. In this study, we identified three different sub-genotypes (B4, B5, and B6) of the B genotype in tick samples. Moreover, we used an unclassified human strain (MG737182) with a previously reported strain, belonging to the B4 genotype of the Gokseong-18 strain. B5 genotypes were only detected in the Gochang region, and the B4 and B6 genotypes were only detected in the Gokseong region. A total of four different B genotypes were detected in the Gokseong region, which is located in the southwestern area of Korea and forms a basin. The collection point of the Gokseong region is located in the Sobaek mountains and is surrounded by Jiri and Mudeung Mountain National Park and Jogye Mountain Provincial Park. Thus, the aggregation and contact with different varieties of wildlife with the tick population may affect the genetic diversity of SFTSV. This correlates to other regions of this study only reporting one strain. In another study, the Gokseong region also had the highest MIR of SFTSV in ticks [21].

Overall, we surveyed the geographical and temporal distribution of nationwide tick populations and performed a molecular and phylogenetic analysis of SFTSV. We identified widely distributed tick species and a high degree of diversity in the SFTSV strains in ticks from different geographical regions. The seasonal distribution of the collected ticks in this study could reflect their life cycle; the peak transmission in summer and autumn may be attributable to the seasonal life cycle of ticks and the increased rates of human outdoor activity. These results show that people must be aware of the risk of exposure to ticks harboring SFTSV during outdoor activities. Further geographical and ecological studies on ticks will improve our understanding of SFTS risks in Korea. The density of tick species varies according to the collection region, season, and year. Thus, further studies are needed to analyze the distribution of ticks and to understand the epidemiology of SFTSV and its risk to public health in Korea.

## Figures and Tables

**Table 1:** Geographical distribution of tick species collected from traps in Korea in 2020.

Region	Number of Ticks Collected (Trap Index (TI))							
	*H. Longi*	*Haemaphysalis* spp.	*H. Flava*	*Ixodes* spp.	*A. Testu*	*I. Nippo*	*H. Japo*	Total
Pocheon	447 (4.7)	72 (0.8)	14 (0.1)	0	0	1 (0.01)	0	534 (5.6)
Inje	1140 (11.9)	638 (6.6)	20 (0.2)	0	0	3 (0.03)	2 (0.02)	1803 (18.8)
Ganghwa	3696 (38.5)	39 (0.4)	102 (1.1)	0	0	0	0	3837 (40.0)
Samcheok	4616 (48.1)	7176 (74.8)	59 (0.6)	0	0	10 (0.1)	0	11,861 (123.6)
Gwangju	1005 (10.5)	851 (8.9)	23 (0.2)	0	0	0	0	1879 (19.6)
Dangjin	5748 (59.9)	2669 (27.8)	218 (2.3)	445 (4.6)	0	6 (0.06)	0	9086 (94.6)
Boryeong	969 (10.1)	500 (5.2)	156 (1.6)	0	1 (0.01)	1 (0.01)	0	1627 (16.9)
Boeun	527 (5.5)	180 (1.9)	50 (0.5)	0	0	1 (0.01)	0	758 (7.9)
Andong	639 (6.7)	28 (0.3)	78 (0.8)	0	0	7 (0.07)	0	752 (7.8)
Sangju	2400 (25.0)	1359 (14.2)	13 (0.1)	1 (0.01)	0	2 (0.02)	0	3775 (39.3)
Gochang	3301 (34.4)	116 (1.2)	122 (1.3)	2 (0.02)	42 (0.4)	0	0	3583 (37.3)
Ulju	2589 (27.0)	2790 (29.1)	23 (0.2)	0	0	8 (0.08)	0	5410 (56.4)
Gokseong	1455 (15.2)	625 (6.5)	38 (0.4)	0	9 (0.1)	9 (0.09)	0	2136 (22.3)
Boseong	186 (1.9)	1458 (15.2)	52 (0.5)	0	1 (0.01)	0	0	1697 (17.7)
Jinju	5978 (62.3)	1990 (20.7)	28 (0.3)	0	130 (1.4)	15 (0.16)	0	8141 (84.8)
Jeju	1247 (13.0)	5197 (54.1)	53 (0.6)	0	0	0	0	6497 (67.7)
Total	35,943 (23.4)	25,688 (16.7)	1049 (0.7)	448 (0.3)	183 (0.1)	63 (0.04)	2 (0.001)	63,376 (41.3)

Trap index (TI), number of collected ticks/number of installed traps; H. longi, Haemaphysalis longicornis; H. flava, Haemaphysalis flava; I. nippo, Ixodes nipponensis; A. testu, Amblyomma testudinarium; H. japo, Haemaphysalis japonica.

**Table 2:** Temporal distribution of ticks and SFTSV infection in Korea in 2020.

Ticks	Stage	Number of Collected Ticks								
		Apr.	May	Jun.	Jul.	Aug.	Sep.	Oct.	Nov.	Total
*H. longi*	Female	556	1601	2683	2955	770	49	12	8	8634
	Male	311	837	813	753	98	40	7	1	2860
	Nymph	4645	11,487	5412	1864	565	430	21	25	24,449
	subtotal	5512	13,925	8908	5572	1433	519	40	34	35,943
*H. flava*	Female	29	15	5	1	8	81	52	56	247
	Male	9	7	2	5	26	95	41	37	222
	Nymph	75	64	205	52	13	47	57	67	580
	subtotal	113	86	212	58	47	223	150	160	1049
*I. nippo*	Female	4	5	0	1	0	1	5	10	26
	Male	0	4	0	1	0	1	2	8	16
	Nymph	5	6	2	1	3	0	4	0	21
	subtotal	9	15	2	3	3	2	11	18	63
*A. testu*	Female	1	3	0	2	1	1	0	0	8
	Male	0	1	0	0	0	0	0	0	1
	Nymph	57	85	16	8	3	2	3	0	174
	subtotal	58	89	16	10	4	3	3	0	183
*H. japo*	Nymph	1	1	0	0	0	0	0	0	2
*H.* spp.	Larva	146	255	212	887	9229	12,517	1819	623	25,688
*I.* spp.	Larva	0	0	0	433	14	1	0	0	448
Total	Female	590	1624	2688	2959	779	132	69	74	8915
	Male	320	849	815	759	124	136	50	46	3099
	Nymph	4783	11,643	5635	1925	584	479	85	92	25,226
	Larva	146	255	212	1320	9243	12,518	1819	623	26,136
	Total	5839	14,371	9350	6963	10,730	13,265	2023	835	63,376

*H. longi, Haemaphysalis longicornis, H. flava, Haemaphysalis flava, I. nippo, Ixodes nipponensis; A. testu, Amblyomma testudinarium; H. japo, Haemaphysalis japonica*; *H.* spp., *Haemaphysalis* spp.; *I.* spp., *Ixodes* spp.

**Table 3:** SFTSV infection in ticks in various regions in Korea in 2020.

Regions.	Number of Collected Ticks	Trap Index	Number of Tested Ticks	Number of Pools	Number of SFTS-Positive Tick Pools	MIR (%) of Ticks
Pocheon	534	5.6	288	92	0	0
Inje	1803	18.8	920	111	0	0
Ganghwa	3837	40.0	1945	271	0	0
Samcheok	11,861	123.6	5944	284	1	0.02
Gwangju	1879	19.6	961	119	18	1.9
Dangjin	9086	94.6	4582	438	0	0
Boryeong	1627	16.9	829	114	0	0
Boeun	758	7.9	411	122	0	0
Andong	752	7.8	399	99	16	4.0
Sangju	3775	39.3	1887	157	0	0
Gochang	3583	37.3	1825	232	10	0.5
Ulju	5410	56.4	2693	207	0	0
Gokseong	2136	22.3	1104	135	18	1.6
Boseong	1697	17.7	870	79	0	0
Jinju	8141	84.8	4106	335	0	0
Jeju	6497	67.7	3288	178	0	0
Total	63,376	41.3	32,052	2973	63	0.2

Trap index, number of collected ticks/ number of installed traps; MIR, minimum infection rate (number of positive pools of ticks/total number of ticks tested × 100).

## Data Availability

Data supporting the conclusions of this article are included within the article. The newly generated sequences were submitted to the GenBank database under the accession numbers MZ423234-MZ423296. The datasets used and/or analyzed during the present study are available from the corresponding author upon reasonable request.
